# Supplementary material for: Dendritic branch structure compartmentalizes voltage-dependent calcium influx in cortical layer 2/3 pyramidal cells
Source: eLife. 2022 Mar 23;11:e76993. doi: 10.7554/eLife.76993 (PMC8979587; doi:10.7554/eLife.76993)
Supplement: Transparent reporting form [file elife-76993-transrepform1.docx]

***eLife’s* transparent reporting form**

We encourage authors to provide detailed information *within their submission* to facilitate the interpretation and replication of experiments. Authors can upload supporting documentation to indicate the use of appropriate reporting guidelines for health-related research (see [EQUATOR Network](http://www.equator-network.org/%20)), life science research (see the [BioSharing Information Resource](https://biosharing.org/)), or the [ARRIVE guidelines](http://www.plosbiology.org/article/info:doi/10.1371/journal.pbio.1000412) for reporting work involving animal research. Where applicable, authors should refer to any relevant reporting standards documents in this form.

If you have any questions, please consult our Journal Policies and/or contact us: [editorial@elifesciences.org](mailto:editorial@elifesciences.org).

**Sample-size estimation**

- You should state whether an appropriate sample size was computed when the study was being designed
- You should state the statistical method of sample size computation and any required assumptions
- If no explicit power analysis was used, you should describe how you decided what sample (replicate) size (number) to use

Please outline where this information can be found within the submission (e.g., sections or figure legends), or explain why this information doesn’t apply to your submission:

We did not perform explicit power analyses, however, our replicate numbers were thoughtfully determined. See below.

**Replicates**

- You should report how often each experiment was performed
- You should include a definition of biological versus technical replication
- The data obtained should be provided and sufficient information should be provided to indicate the number of independent biological and/or technical replicates
- If you encountered any outliers, you should describe how these were handled
- Criteria for exclusion/inclusion of data should be clearly stated
- High-throughput sequence data should be uploaded before submission, with a private link for reviewers provided (these are available from both GEO and ArrayExpress)

Please outline where this information can be found within the submission (e.g., sections or figure legends), or explain why this information doesn’t apply to your submission:

For all individual experiments, we performed >3 trials of calcium imaging for each ROI. The calcium signals we observed were extremely consistent across trials, so the primary noise source is optical shot noise, and >3 is more than enough for replicable data.

Data in Figure 2 and Figure 3A-B was collected during an exploratory phase of research, and so did not involve premeditated sample size computation.

Experiments in Figures 3C-H, Figure 4, and Figure 5 relied upon estimating correlations between calcium influx evoked by 1 action potential and some other signal we measured (different for each figure). From Figure 2, we knew what the distribution of calcium influx evoked by 1 action potential was within the distance range (>80 µm from the soma) was. Therefore, we collected data until we had at least 3 data points from each quartile, but preferentially more (experiments in Figures 4 & 5 were extremely challenging).

For experiments in Figure 6, we designed an experiment that depended on comparison of pairs of recording sites within cells. Because each cell may have different properties, we decided to acquire at least 8 cells, with >1 pair per cell. Experiments of this kind often use just 3-4 cells to make conclusions, so we wanted to exceed the minimum and acquire a robust dataset.

All other experiments were computational simulations and as such do not contain sample variance.

**Statistical reporting**

- Statistical analysis methods should be described and justified
- Raw data should be presented in figures whenever informative to do so (typically when N per group is less than 10)
- For each experiment, you should identify the statistical tests used, exact values of N, definitions of center, methods of multiple test correction, and dispersion and precision measures (e.g., mean, median, SD, SEM, confidence intervals; and, for the major substantive results, a measure of effect size (e.g., Pearson's r, Cohen's d)
- Report exact p-values wherever possible alongside the summary statistics and 95% confidence intervals. These should be reported for all key questions and not only when the p-value is less than 0.05.

Please outline where this information can be found within the submission (e.g., sections or figure legends), or explain why this information doesn’t apply to your submission:

In almost every case, our data revealed extremely strong trends. We performed statistical tests on correlations and regressions but refrained from comparing between the high/low calcium populations because these were for visualization. In Figure 6, the we perform direct comparisons between high/low calcium populations because they were carefully selected to be distance-matched pairs from within each cell.

(For large datasets, or papers with a very large number of statistical tests, you may upload a single table file with tests, Ns, etc., with reference to sections in the manuscript.)

**Group allocation**

- Indicate how samples were allocated into experimental groups (in the case of clinical studies, please specify allocation to treatment method); if randomization was used, please also state if restricted randomization was applied
- Indicate if masking was used during group allocation, data collection and/or data analysis

Please outline where this information can be found within the submission (e.g., sections or figure legends), or explain why this information doesn’t apply to your submission:

We split data into high and low Ca groups to facilitate visualization, however, all our relevant results are presented as group data in scatter plots.

**Additional data files (“source data”)**

- We encourage you to upload relevant additional data files, such as numerical data that are represented as a graph in a figure, or as a summary table
- Where provided, these should be in the most useful format, and they can be uploaded as “Source data” files linked to a main figure or table
- Include model definition files including the full list of parameters used
- Include code used for data analysis (e.g., R, MatLab)
- Avoid stating that data files are “available upon request”

Please indicate the figures or tables for which source data files have been provided:

We will make computational simulations performed in Figures 7 & 8 available on modeldb, and are in the process of preparing them for submission to that database. All data is available on Harvard Dataverse at doi:10.7910/DVN/ZHNKGE.
